# Evaluation of Alternative Halo Ring Positions in Children Using Tomography

**DOI:** 10.6061/clinics/2019/e781

**Published:** 2019-03-05

**Authors:** Mauro Costa Morais Tavares-Júnior, Diego Ubrig Munhoz, João Paço Vaz de Souza, Raphael Martus Marcon, Alexandre Fogaça Cristante, Olavo Biraghi Letaif

**Affiliations:** I Departamento de Ortopedia e Traumatologia, Instituto de Ortopedia e Traumatologia (IOT), Hospital das Clinicas HCFMUSP, Faculdade de Medicina, Universidade de Sao Paulo, Sao Paulo, SP, BR; II Grupo de Coluna, Departamento de Ortopedia e Traumatologia, Instituto de Ortopedia e Traumatologia (IOT), Hospital das Clinicas HCFMUSP, Faculdade de Medicina, Universidade de Sao Paulo, Sao Paulo, SP, BR

**Keywords:** Halo Ring, Tomography, Morphology

## Abstract

**OBJECTIVES:**

The halo ring can be applied in children, through skeletal traction or a halo vest device, to treat many cervical spine pathologies, including traumatic injuries and pathologies related to deformities. However, the procedure is associated with various complications, such as infection, pin loosening, and respiratory and neurological problems. Although widely studied in adults, the best pin insertion site in children and the correlations of pin insertion sites with outcomes and complications have not been completely elucidated. This study aimed to determine alternative pin placement sites based on a morphological analysis of the infant skull by computerized tomography (CT).

**METHODS:**

An analytical-descriptive study was performed using 50 CT scans from children. The Wilcoxon and Friedman tests were used.

**RESULTS:**

A linear and directly proportional relation was found between cranial thickness and patient age. The average thicknesses of the anterior points across all ages analyzed ranged from 4.16 mm to 4.98 mm. The thicknesses of the posterior points varied from 3.94 mm to 4.27 mm. Within each age range, points 1 cm above the standard insertion sites had thicknesses similar to those of the standard sites, and points 2 cm above the standard insertion sites had thicknesses greater than those of the standard sites.

**CONCLUSIONS:**

The cranial thickness at all points increases linearly with age. Points 1 and 2 cm above the standard insertion sites are viable alternatives for the placement of halo pins. Preoperative CT can aid in choosing the best positioning sites for pins in the skull.

## INTRODUCTION

The skeletal halo ring device is the stiffest cervical immobilization available 1 for various cervical spine pathologies. The first described use of the halo in children was as a high cervical stabilization method after cervical arthrodesis in patients with poliomyelitis 2. The use of the halo in children has been expanded; it can be applied to treat spine fractures 3, although further investigations of the eligibility criteria related to fracture patterns are needed, and the halo can also be applied with preoperative gravitational traction as an adjunct method for the treatment of skeletal deformities 4-7.

As the indications for use of the halo have increased, possible complications associated with its use have been noted, with the frequency of general complications, such as pin infection (more common among children older than 11 years of age), pin loosening (more common in children younger than 10 years of age) 8, respiratory difficulty, falls 9, cranial nerve paralysis, bradycardia and aesthetic changes related to scars, reaching 53% 10.

For better results after traction and immobilization and to reduce the risk of complications, some studies have analyzed the best position for the insertion of halo pins. Relatively safe sites for the insertion of pins are described as the anterior zone 1 cm above the orbital edge of the transition between the middle and lateral thirds, the posterior zone located diagonally opposed to the contralateral anterior pin and 1 cm above the ear spirals 1,11 (primarily in adults but not yet completely established in children due to skeletal differences caused by incomplete skeletal development in children). Previous studies have recommended routine tomography for halo ring planning due to significant differences in the thicknesses of the various areas of the skull at different ages 12-14. Based on the standard anterolateral and posterolateral positions of the pins, differences were identified between the left and the right sides of the posterior area, but not the anterior area. No significant differences related to gender or ethnicity have been reported 15.

There is currently no consensus in the literature regarding the best positioning of the halo in children. This study aimed to determine alternative pin placement sites based on a morphological analysis of the infant skull by computerized tomography (CT).

## MATERIALS AND METHODS

In this observational descriptive analytical study, 50 cranial CT scans were analyzed from patients at a university hospital. The examinations were performed in 2016.

The inclusion criteria allowed for the inclusion of patients between 3 and 17 years of age of both genders who were subjected to cranial CT, which could be reconstructed in 3D, for reasons unrelated to trauma.

The exclusion criteria were patients with incomplete registry entries related to demographic data, CT scans that could not be reconstructed in 3D, cranial trauma that could have caused bone deformities, invasive surgical procedures, congenital malformations, cranial deformities (secondary to other pathologies, such as thalassemia), sickle-cell anemia and osteoporosis, and oncologic diseases with cranial osseous metastasis or osseous mineralization impairment (e.g., multiple myeloma). The exclusion criteria aimed to guarantee that patients with anatomic alterations were not selected.

The following parameters were evaluated in this study: patient age (measured in years), gender (male or female) and skull cap thickness (in millimeters) at the 4 standard reference points and at 1 cm and 2 cm above these points. The patients were divided into 3 groups according to their age range: G1 (3-5 years of age), G2 (6-12 years of age) and G3 (13-17 years of age). This division of groups by age is supported in the literature 16, as the cranial morphologies of children within such age ranges are similar.

Measurements were obtained by two different examiners. 3D reconstruction of CT was performed using ISite^®^ Enterprise software (Philips, Amsterdam, The Netherlands). The results obtained after statistical analysis are represented as graphs, charts, tables or schematics.

The data were stored in an Excel^®^ for Mac spreadsheet (Microsoft Corporation, Redmond, Washington), and after checking the entries, the data were exported to SPSS^®^ 23 for Mac (IBM company, Armonk, New York) for statistical analysis. In the descriptive statistics analysis, continuous data were reported as the mean and standard deviation (SD). Categorical data were reported as absolute frequencies and the respective categorical proportions. Inferential statistical analysis was performed to compare sides and the various pin insertion sites in relation to the cranial thickness measurements obtained by tomography. The normality of the data distribution was tested; as a normal distribution was not observed, nonparametric tests of paired comparisons were utilized (Wilcoxon and Friedman tests). Differences for which *p*<0.05 were accepted as statistically significant differences.

**Figure 1:**
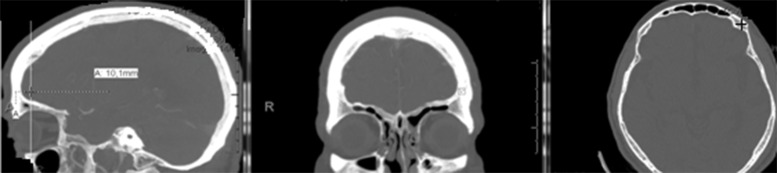
Site of pin insertion via recognition line mode viewed in the sagittal cut and via locating mode viewed in the coronal cut. Images were generated by the software for the measurements of both internal and external border thickness, as shown by the cross in the axial cut.

**Figure 2:**
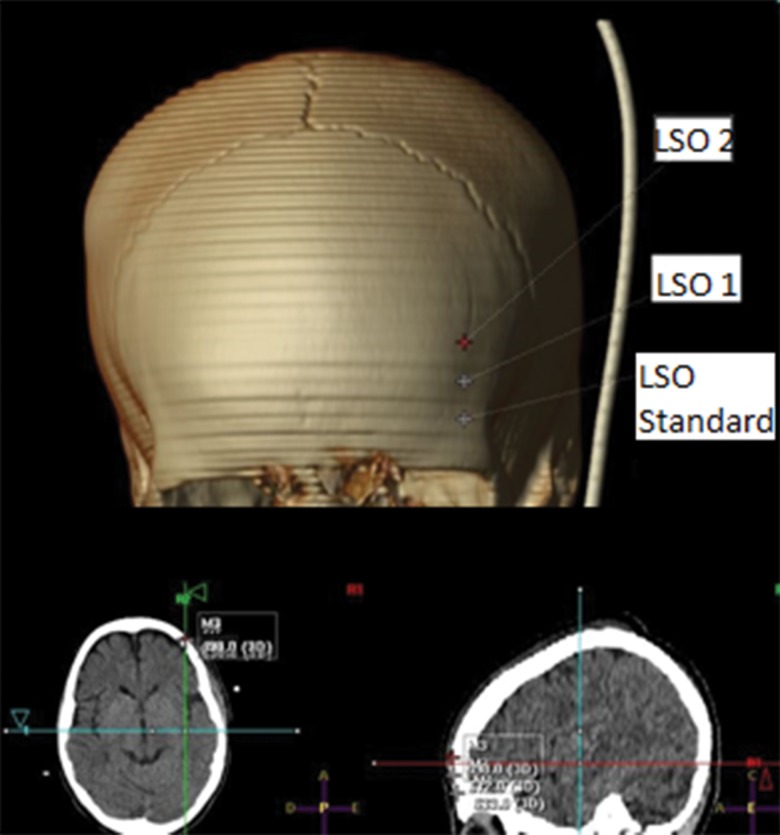
Example of the sites for the standard LSO, LSO 1 and LSO 2 points.

**Figure 3:**
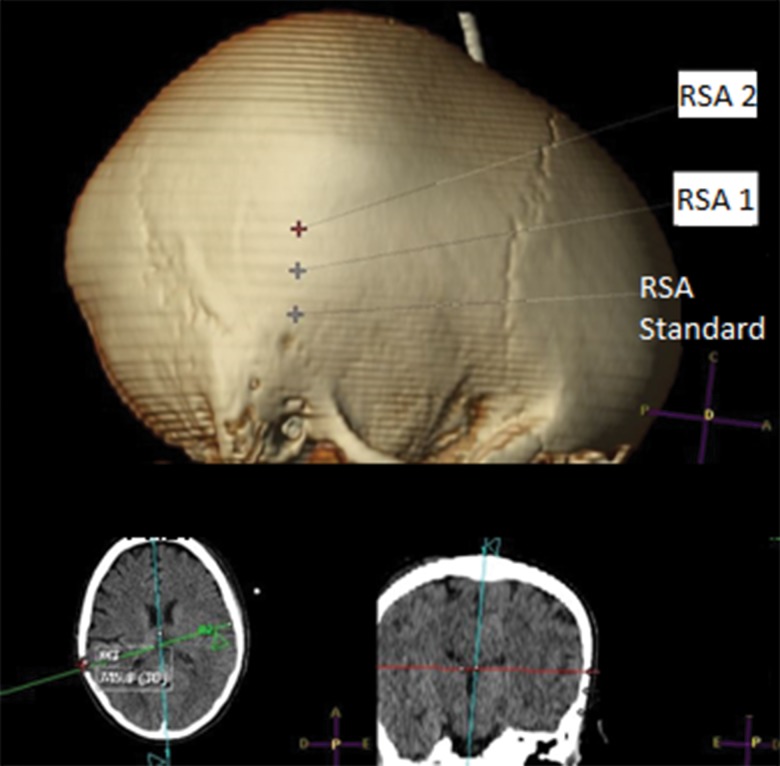
Example of the sites for the standard RSA, RSA 1 and RSA 2 points.

The evaluated pin insertion points were measured for the 50 tomographic examinations selected using private software through an osseous window in sagittal, coronal and axial cuts (Figures 1-3). Measurements were obtained through axial cuts, coronal cuts, and sagittal cuts, and 3D reconstruction was used as a guide to locate the necessary points to minimize measurement errors.

### Ethics

This study obtained ethics committee approval, and patient consent was not required.

## RESULTS

In this study, CT scans from 50 patients (26 male and 24 female) were analyzed. The average age among all 3 age groups was 9.52 years, with an SD of 4.53 years. The average male age was 10.08 years (SD: 4.07), and the average female age was 8.92 years (SD: 5.00).

The evaluation consisted of 9 patients in group 1, 26 patients in group 2 and 15 patients in group 3. Among the male patients, 3 belonged to group 1 (11.5%), 16 belonged to group 2 (61.5%) and 7 belonged to group 3 (36.9%). Among the female patients, 6 belonged to group 1 (25%), 10 belonged to group 2 (41.7%) and 8 belonged to group 3 (33.3%). The average age of group 1 was 3.3 years (SD: 0.50), the average age of group 2 was 8.42 years (SD: 2.32) and the average age of group 3 was 15.12 years (SD: 1.25).

The cranial thickness values in the comparative analysis among groups are given in Tables 1-4.

The figures reveal a linear relation between age and the thickness measured at each reference point.

In comparing skull cap thickness within a single group, we assumed symmetry of the left and right sides, and an average was used for statistical analysis. Thus, the following thicknesses were obtained: G1: standard supra-auricular (SA) average of 3.20 (SD: 0.89), SA1 average of 3.45 mm (SD: 1.15), and SA2 average of 3.22 mm (SD: 0.94); G2: standard SA average of 4.03 mm (SD 1.01), SA1 average of 4.15 mm (SD 1.26), and SA2 average of 4.25 mm (SD 1.03); and G3: standard SA average of 4.30 mm (SD 0.97), SA1 average of 4.47 mm (SD 0.99), and SA2 average of 4.68 mm (SD 1.08).

For the supraorbital pins, the following thicknesses were obtained: G1: standard supraorbital (SO) average of 3.63 mm (SD 0.89), SO1 average of 3.78 mm (SD 0.83), and SO2 average of 4.04 mm (SD: 0.79); G2: standard SO average of 3.93 mm (SD 1.25), SO1 average of 4.05 mm (SD 1.18), and SO2 average of 4.64 mm (SD 1.30); and G3: standard SO average of 4.97 mm (SD 1.67), SO1 average of 5.08 mm (SD 1.28), and SO2 average of 6.07 mm (SD 1.29) (Charts 22 and 24).

## DISCUSSION

In the scientific literature on infant skull morphology, the best locations for the insertion of halo ring pins have not been determined. Some studies suggest performing cranial tomography before conducting the halo ring procedure 12,13, while other studies have confirmed the more frequently used sites for anterolateral and posterolateral pins 16,17.

Upon analyzing the collected data, we observed a linear increase in infant cranial thickness with increasing age for all points. This finding was similar to previous results reported in the literature 16,18. Thus, it is important to plan for the positioning of the halo ring pins according to age because the skull cap thickness is not uniform across the entire surface area of the skull, which can lead to potential complications of the procedure.

We found average thicknesses for the anterior pins of 4.01 mm (RSO) and 3.86 mm (LSO) in children between 5 and 12 years of age. Considering only the anterolateral normal standard, Garfin et al. 16 found an average thickness of 6.1 mm for this same group. Regarding the posterior pins, we found average thicknesses of 4.07 mm (LSA) and 3.99 mm (RSA), while Garfin et al. 16 reported an average of 5.9 mm for the posterolateral standard. The thicknesses found in the present study were smaller than the thicknesses cited in the literature, which may be explained by the smaller stature and biotype of the Brazilian population 19. Another possible explanation for the differences in these values may be that the quality of the imaging used in examinations has improved with recent technology. The measurement error may be smaller in light of higher resolution and more precise tomographic cuts. Another possibility is that the measurement error itself differs due to different techniques or skill levels of the evaluators who performed the measurements or differences in the sizes of the samples, which may significantly alter the average values and the SD.

Regarding measurements in children younger than 6 years of age, Letts et al. 14. reported values that ranged from 1.1 to 4.3 mm, and in the present study, all the thickness measurements of children younger than 6 years of age ranged from 3.12 to 4.08 mm. The present work shows a smaller variation in thickness values based on CT scans with thinner cuts, which are easier to evaluate when utilizing 3D reconstructions. Another possible explanation is the size of the sample, as larger samples theoretically correspond to smaller confidence intervals.

Considering the areas of pin insertion, statistically significant differences were found between the LSA2 of group 3 and the LSA2 of group 1, revealing a greater skull cap thickness in older children. Only the left side reached statistical significance, which may be due to anatomical alterations in the studied population or an insufficient number of patients to determine differences on the right side. Regarding skull cap thickness, at the standard points, 1 cm above the standard points and 2 cm above the standard points, we observed a linear increase in skull cap thickness within each age range.

In the present study, we revealed that there are significant differences in skull cap thickness at supraorbital points within each age group for comparisons of standard positioning *vs*. 1 cm above standard positioning *vs*. 2 cm above standard positioning, with a progressive increase in ossification. However, we cannot confirm that these points are better than the standard points, as no biomechanical study has proven their superiority to date. These data only reveal alternative points that display greater osseous thickness on CT.

Placement of the halo ring in children is a relatively safe procedure. It is often not necessary to perform CT examinations prior to pin insertion. However, prior CT exams may facilitate better positioning of the pins, especially in more complex cases in which the local osseous anatomy exhibits any clinical alterations. Therefore, the use of CT with 3D reconstruction is justified in planning the procedure.

The limitations of the present study include its retrospective design and the fact that the individuals in the various age ranges were unevenly distributed, as some age ranges were more represented than others. Due to the specificity of the studied population, caution should be used when applying these results to external populations.

The advantages of this study include the use of CT scans with thin cuts and 3D reconstruction to minimize measurement errors, the use of independent statistics for data analysis, and the use of updated software for statistical analysis. Precision in the analysis of osseous thickness measurements of the skull cap allows safer insertion of halo rings, minimizing possible complications.

Notably, the present study pioneered a joint analysis of different points on the skull cap by employing CT scans. CT examination may be a useful and desirable tool for refining operative planning.

Additional studies, ideally with larger sample sizes, are necessary to increase our understanding of both cranial development and cranial morphology. Studies of cranial morphology according to specific pathologies related to osseous formation and development are also important for developing safer and more precise treatments.

## CONCLUSION

Children exhibit an increase in skull cap thickness with age, which was revealed by comparing children of different ages. Alternative points for pin insertion at 1 and 2 cm above the classic locations for halo installation were shown to be viable and safe upon comparative analysis using CT examinations.

## AUTHOR CONTRIBUTIONS

Tavares-Júnior MC, Munhoz DU, Souza JP reviewed the literature, designed the project, collected and analyzed the data, and wrote the manuscript. Marcon RM and Cristante AF were responsible for the final review of the literature and project, designed the study, analyzed the data and reviewed the final version of the manuscript. Leitaf OB designed the study, analyzed the data and performed the final review of the manuscript.

## Figures and Tables

**Table 1:** Supra-auricular pin comparison.

-Supra-auricular pin comparison between children 3 and 5 years of age		Statistics*
Standard Supra-Auricular	Average (mm) Standard Deviation	3.20 0.89
Supra-Auricular_1 cm	Average Standard Deviation	3.45 1.15
Supra-Auricular_2 cm	Average Standard Deviation	3.22 0.94

-Supra-auricular pin comparison between children 6 and 12 years of age		Statistics^†^
Standard Supra-Auricular	Average (mm) Standard Deviation	4.03 1.01
Supra-Auricular_1 cm	Average Standard Deviation	4.15 1.26
Supra-Auricular_2 cm	Average Standard Deviation	4.25 1.03

-Supra-auricular pin comparison between children 1 and 17 years of age		Statistics^‡^
Standard Supra-Auricular	Average (mm) Standard Deviation	4.30 0.97
Supra-Auricular_1 cm	Average Standard Deviation	4.47 0.99
Supra-Auricular_2 cm	Average Standard Deviation	4.68 1.08

^*^No significant differences were observed among the points (Friedman test: *p*=0.35).

^†^No significant differences were observed among the points (Friedman test: *p*=0.52).

^‡^No significant differences were observed among the points (Friedman test: *p*=0.15).

**Table 2:** Supraorbital pin comparison between children 3 and 5 years of age.

		Statistics*			
Standard Supraorbital	Average (mm) Standard Deviation	3.63 0.89			
Supraorbital_1 cm	Average Standard Deviation	3.78 0.83			
Supraorbital_2 cm	Average Standard Deviation	4.04 0.79			

	Supraorbital_1 cm *vs.* Standard Supraorbital	Supraorbital_2 cm *vs.* Supraorbital_1 cm^†^	Supraorbital_2 cm *vs.* Standard_Supraorbital^†^
Z	-1.175^c^	-2.136^c^	-2.958^c^
Asymptotic Significance (Bilateral)	0.24	0.033	0.003

^*^Wilcoxon statistical test.

^†^Significant differences were observed for 1 cm *vs*. 2 cm and for standard *vs*. 2 cm.

**Table 3:** Supraorbital pin comparison between children 6 and 12 years of age.

		Statistics*			
Standard Supraorbital	Average (mm) Standard Deviation	3.93 1.25			
Supraorbital_1 cm	Average Standard Deviation	4.05 1.18			
Supraorbital_2 cm	Average Standard Deviation	4.64 1.30			

	Supraorbital_1 cm *vs.* Standard Supraorbital	Supraorbital_2 cm *vs.* Supraorbital_1 cm^†^	Supraorbital_2 cm *vs.* Standard_Supraorbital^†^
Z	-1.160^c^	-4.232^c^	-4.488^c^
Asymptotic Significance (Bilateral)	0.24	<0.001	<0.001

^*^Wilcoxon statistical test.

^†^Significant differences were observed for 1 cm *vs*. 2 cm and for standard *vs*. 2 cm.

**Table 4:** Supraorbital pin comparison between children 12 and 17 years of age.

		Statistics*			
Standard Supraorbital	Average (mm) Standard Deviation	4.97 1.67			
Supraorbital_1 cm	Average Standard Deviation	5.08 1.28			
Supraorbital_2 cm	Average Standard Deviation	6.07 1.29			

	Supraorbital_1 cm *vs.* Standard Supraorbital	Supraorbital_2 cm *vs.* Supraorbital_1 cm^†^	Supraorbital_2 cm *vs.* Standard_Supraorbital^†^
Z	-0.545^c^	-4.284^c^	-3.493^c^
Asymptotic Significance (Bilateral)	0.586	<0.001	<0.001

^*^Wilcoxon statistical test.

^†^Significant differences were observed for 1 cm *vs*. 2 cm and for standard *vs*. 2 cm.
